# Two macrophages, osteoclasts and microglia: from development to pleiotropy

**DOI:** 10.1038/s41413-020-00134-w

**Published:** 2021-02-10

**Authors:** Ji-Won Lee, In-Hee Lee, Tadahiro Iimura, Sek Won Kong

**Affiliations:** 1 Department of Nephrology, Transplant Research Program, Boston Children’s Hospital, Boston, MA 02115 USA; 2 Department of Pharmacology, Graduate School of Dental Medicine, Hokkaido University, Sapporo, 060-8586 Japan; 3 Computational Health Informatics Program, Boston Children’s Hospital, Boston, MA 02115 USA; 4 Department of Pediatrics, Harvard Medical School, Boston, MA 02115 USA

**Keywords:** Bone, Neurophysiology

## Abstract

Tissue-resident macrophages are highly specialized to their tissue-specific microenvironments, activated by various inflammatory signals and modulated by genetic and environmental factors. Osteoclasts and microglia are distinct tissue-resident cells of the macrophage lineage in bone and brain that are responsible for pathological changes in osteoporosis and Alzheimer’s disease (AD), respectively. Osteoporosis is more frequently observed in individuals with AD compared to the prevalence in general population. Diagnosis of AD is often delayed until underlying pathophysiological changes progress and cause irreversible damages in structure and function of brain. As such earlier diagnosis and intervention of individuals at higher risk would be indispensable to modify clinical courses. Pleiotropy is the phenomenon that a genetic variant affects multiple traits and the genetic correlation between two traits could suggest a shared molecular mechanism. In this review, we discuss that the Pyk2-mediated actin polymerization pathway in osteoclasts and microglia in bone and brain, respectively, is the horizontal pleiotropic mediator of shared risk factors for osteoporosis and AD.

## Introduction

Bone undergoes constant remodeling to maintain bone homeostasis between bone formation and resorption by osteoblasts and osteoclasts, respectively.^1^ Bone remodeling is a process of bone resorption followed by replacement of new bone formation, which is a tightly balanced work referred to as coupling.^2^ The osteoclast is a large multinucleated cell derived from monocyte/macrophage lineage of pluripotential hematopoietic stem cells (HSCs). Osteoclasts degrade the bone matrix with hydrogen ions and catalytic enzymes, whereas osteoblasts—mononuclear cells derived from mesenchymal cells—secrete organic matrix molecules that contribute to the formation of new bone. Osteoclasts undergo differentiation and fusion resulting in multinucleated cells, in the presence of macrophage colony-stimulating factor (M-CSF, also known as CSF1) and the key osteoclastogenic cytokine, receptor activator of NF-kB ligand (RANKL), characterized by expression of osteoclast markers, such as tartrate-resistant acid phosphatase,^3^ matrix metalloproteinase (MMP-9),^4^ cathepsin K^5^, and vacuolar [H^+^]-ATPase.^6^ Bone resorption is a necessary process for bone growth, tooth eruption, fracture healing and maintaining appropriate level of calcium in blood. Under pathological conditions such as estrogen deficiency or inflammatory conditions like rheumatoid arthritis, abnormal osteoclast proliferation, and differentiation accelerate bone resorption that results in osteolysis.^1^

Osteoporosis is a systemic disorder characterized by abnormally increased osteoclasts activity, therefore leading to bone fragility and an increased risk of fracture, and current therapies are targeting inhibition of osteoclast differentiation and function.^7,8^ This imbalance between resorption and formation is induced by diverse risk factors such as alteration in hormone expression, nutrition, mobility, and senescence. Additionally, some rare genetic disorders show decreased bone resorption that leads to osteopetrosis.^9,10^

Microglia are innate immune cells in the central nervous system (CNS) that account for 10%–15% of all cells in the human brain. Microglia are specialized brain-resident macrophages whose functions in the brain include phagocytosis and provision of trophic support.^11,12^ Besides, these cells are active regulators of synapse formation, plasticity, and elimination.^13^ Microglia engulf C1q—the initiating protein of classical complement pathway—tagged synapses in developing brain^12^ and seem to be responsible for synaptic loss in neurodegenerative disorders. As such dysregulation of microglia has been found in diverse neuropsychiatric, neurodegenerative, and neuroinflammatory diseases.^14^

Alzheimer’s disease (AD) is the most common neurodegenerative disorder that is likely affecting over 40 million patients worldwide. AD is also the most common form of dementia that accounts for more than 60% of sporadic cases. Our understanding of AD pathobiology has been greatly improved over the past two decades; however, no disease-modifying treatment is available for individuals diagnosed with AD.^15^ Age is the primary risk factor for AD and an accumulation of misfolded/aggregated proteins such as senile plaques and neurofibrillary tangles (NFTs) cause pathological changes in brain.^16^ The amyloid β peptides (Aβ) are natural cleavage products of the Aβ precursor protein (APP) by β- and γ-secretases and aggregate to form oligomers and fibrils. Of Aβ peptides, Aβ_42_ (i.e., 42 amino acids long cleavage product of APP) is aggregation-prone and more immunogenic compared to the other isoforms such as Aβ_40._ Brain is the most studied organ for APP expression and function; however, the other splicing isoforms of APP are expressed in many other tissues such as skin, heart, muscle, adipose tissue, liver, spleen, skin, and intestine.^17^ Soluble Aβ can be transported across the blood–brain barrier, from blood to brain via the receptor for advanced glycation end-products (RAGE), and from brain to blood via low-density lipoprotein receptor-related protein 1 (LRP1). Interestingly, Aβ_42_ can enhance osteoclast differentiation and activation.^18^ Aβ deposition is less correlated with cognitive decline compared to accumulation of NFTs due to hyperphosphorylated forms of tau. Multiple evidence support that Aβ accumulation precedes and drives tau aggregation. Nonetheless, Aβ seems to be responsible for synaptic failure and mitochondrial dysfunction that are observed in multiple brain regions in patients with AD. The innate immune system responds to Aβ deposition. In early stage of disease, microglia engulf Aβ through phagocytosis and astrocytes use receptor-mediated internalization to clear Aβ.^19,20^ As the disease progresses, activated microglia release chemokines and cytokines to further facilitate inflammatory reaction that damages neural tissues in later stage of AD.^15^ Moreover, genes with common and rare risk alleles for AD (e.g., *TREM2*^21^) are preferentially or exclusively expressed in microglia. These findings strongly support the role of microglia in the pathogenesis of AD according to the disease stages of AD.^22^ To date, clinical trials targeting Aβ failed to demonstrate clinical efficacy. The Aβ deposition likely begins as early as 20 years before cognitive impairment.^15^ Therefore, early identification of high-risk groups for AD using clinical and molecular biomarkers is highly required.

### Pleiotropic effect of DNA variants associated with bone and brain disorders

Two tissue-resident cells of myeloid origin—i.e., osteoclasts and microglia—contribute the pathogenesis of osteoporosis and AD, respectively.^23,24^ These cell types are specialized to their microenvironment for its main functional role in each tissue, and seem to be disconnected, physically and functionally; however, a rare genetic disorder affecting brain and bone suggests the pleiotropy of a causal gene. Nasu-Hakola disease (NHD) is an autosomal recessive disorder caused by rare genetic variants in either triggering receptor expressed on myeloid cells 2 (*TREM2)* or DNAX Adaptor Protein 12 kD (*DAP12)* genes. NHD is characterized by recurrent bone fractures and progressive presenile dementia.^25^ Osseous symptoms due to osteoporotic lesions start typically in the age of 20–30 years followed by neurologic symptoms such as dementia that are observed in the age of 40–50 years. *DAP12* encodes the tyrosine kinase binding adaptor protein (TYROBP) and *TREM2* encodes the triggering receptor expressed on myeloid cells 2 (TREM2). These genes are the components of a signaling complex involved in the regulation of immune responses, the differentiation of osteoclasts, and in the phagocytic activity of microglia.^23^ The exact mechanism of pathogenesis for NHD is unknown.

Low bone mineral density (BMD) is associated with cognitive decline and a higher risk of AD, and the risk of fracture is increased in patients with AD. As such epidemiological studies suggest associations between AD and osteoporosis^26–28^; however, there is no consistent evidence that osteoporosis is a risk factor of AD at the population-scale. As often observed in individuals with NHD, BMD change may precede cognitive decline in AD due to shared biological pathways that are affected by common genetic and/or environmental risk factors.^29^ Here we describe converging molecular pathways between osteoclasts and microglia that may explain, in part, horizontal pleiotropy of shared genetic risk factor between osteoporosis and AD. Firstly, we review the developmental origins of osteoclasts and microglia. Secondly, the signaling pathways—i.e., TREM2/DAP12, CSF1/CSF1R and CCR5 pathways—that are key regulators of both cell types are summarized. Thirdly, a converging pathway, *PYK2 pathway*, involving actin/microtubule formation for cytoskeletal rearrangement is highlighted. Finally, we discuss the genetic correlation between osteoporosis and AD.

### Developmental perspective: osteoclasts and microglia

**Fig. 1:**
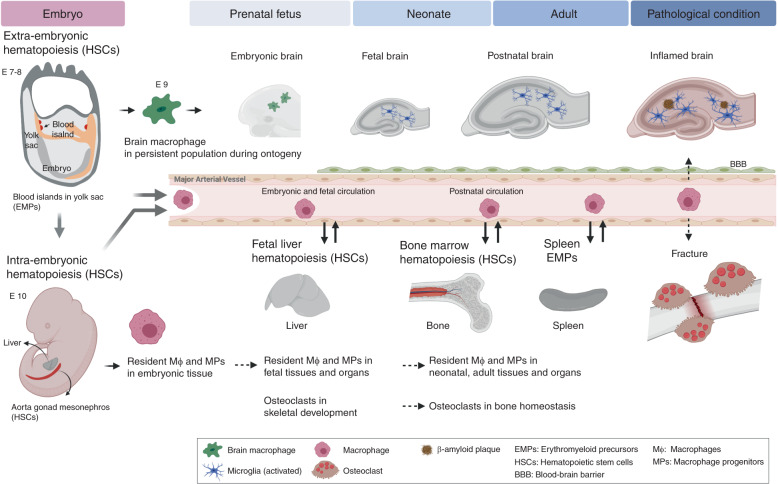
Developmental overview and homeostasis regulations of osteoclasts and microglia. Primitive macrophages exit blood islands of the yolk sac (YS) with the initiation of circulation and colonized the neuroepithelium from E8.5 to give rise to microglia. In a steady state, YS-derived macrophages are differentiated into microglia which, in turn, self-renew throughout ontogeny until adulthood. Under pathological conditions, circulating progenitors or resident macrophages that are allowed to reside in brain contribute to newly differentiated microglia. The migration of definitive brain macrophage is initiated from the HSCs of the embryonic aorta-gonad-mesonephros (AGM). Subsequently, HSCs expand in the fetal liver, which is the main source for tissue-specific macrophages. Osteoclasts originated from resident embryonic-myeloid progenitors (EMPs) lineage, which are long-lived and can participate in postnatal bone maintenance. Adult bone homeostasis is maintained by osteoclast fusion that are derived from EMPs and HSCs supplied by bone marrow and possibly by spleen. In injured skeletal tissues, for instance due to bone fracture, circulating monocytes and macrophages from spleen migrate into damaged tissue and contribute to tissue repairing

Osteoclasts and microglia are tissue-resident and -specific cells of monocyte/macrophage lineage in skeletal and neural systems, respectively.^30,31^ During ontogeny, the initial origin of macrophages is the blood island in yolk sac (YS), in which extraembryonic hematopoiesis takes place at mouse embryonic days E7–8, and 2–3 weeks of human gestation (Fig. 1). These YS-derived cells can produce macrophages, erythrocytes and lymphocytes.^32,33^ The YS-derived macrophages are distributed in later stages of whole embryonic body and primitive organs, in which hematopoiesis is succeeded to intraembryonic tissues which encompasses the aorta, gonads, and mesonephros (AGM) where HSCs are initially generated.^30,31,34^

In adulthood, there are mainly three origins of intraembryonic hematopoiesis: embryonic vessels, fetal liver, and bone marrow. The migration of YS-derived hematopoietic precursors rapidly initiates the intraembryonic hematopoiesis from major arterial vessels in mouse embryos beginning at E10.5 in mice (around 3 weeks of human gestation). This intraembryonic hematopoiesis is definitive to give rise to HSCs with multi-lineage potential. Subsequently HSCs expand in the fetal liver; main site of fetal hematopoiesis that peaks at E16.5 (around 4 weeks human gestation) before transitioning to the bone marrow that becomes the main site of hematopoiesis in adult life. Therefore, prior to the intraembryonic hematopoiesis, embryonic macrophages throughout whole body including brain-specific microglia are originated from extraembryonic YS.^30,31^ In the next step, the fetal liver gives rise to fetal monocytes, which subsequently differentiate and replace fetal and prenatal macrophages except microglia in CNS. Although the bone marrow hematopoiesis is established since perinatal stages, and becomes main source of macrophages in adulthood, it is not still clear the contributions of the embryonic YS- and fetal liver-derived macrophages to the adult tissue-resident macrophages.

The developmental origin of microglia is the blood island developed in embryonic YS at E7-8 in mouse. The YS-derived brain macrophages colonize to the neuroepithelium around E9, and then differentiate into microglia that is maintained throughout ontogeny although brain under pathological conditions like inflammation allow circulating progenitors reside in brain and contribute to newly differentiated microglia^35,36^ (Fig. 1). In bone and mineral homeostasis, it has been dogmatically understood that bone marrow-derived monocytes can be a major population of osteoclast precursors. In fact, osteopetrotic phenotype has been partially treated by bone marrow transplantation in humans and mice, suggesting partial requirement of bone marrow as a source of osteoclasts.^37^ Contribution of circulating osteoclastic precursors has been also reported.^38^ During perinatal skeletal development, osteoclasts and chondroclasts (cartilage resorbing cells) participate in bone modeling, endochondral bone formation, and tooth eruption, all of which are essential process for pre- and postnatal skeletal development. Recent findings showed that embryonic erythromyeloid precursors (EMPs) derived from YS and fetal liver contributed to osteoclasts participated in early skeletogenesis.^37,39^ Adult bone homeostasis is maintained by iterative fusion of osteoclasts derived from the fetal EMPs and HSCs supplied by bone marrow.^39^ Of note, YS-derived osteoclasts can be colonized in the adult spleen and contribute to bone repair after injury.^39^

In terms of differentiation potential hierarchy, dendritic cells (DCs), monocytes and macrophages are understood to share the monocyte-macrophage DC progenitor (MDP) derived from HSCs. More recent studies identified a downstream progenitor cell population from the MDP, namely common monocyte progenitor (cMoP), whose differentiation potential is restricted to monocytes and macrophages.^40^ The cMoP is also identified in human umbilical cord blood and in bone marrow. Although it remains elusive what is the exact population of circulating progenitors that contribute to tissue-specific macrophages both in injured bone and brain, these populations likely share common regulatory pathways in their cellular differentiation.

### Common signaling pathways between osteoclasts and microglia

**Fig. 2:**
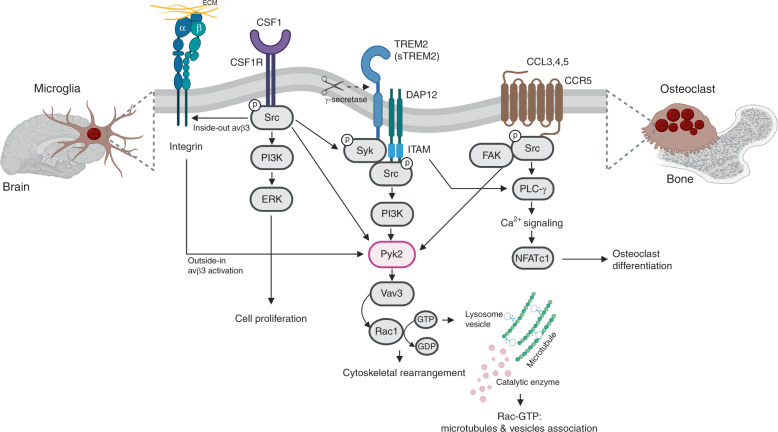
TREM2/DAP12-, CSF1/CSF1R-, and CCR5 pathways in osteoclasts and microglia. Once ligands bind to TREM2, two tyrosine residues in the immunoreceptor tyrosine-based activation motif (ITAM) of DAP12 are phosphorylated, which in turn recruits Syk kinase to activate downstream molecules such as Src tyrosine kinase and phosphatidylinositol 3-kinase (PI3K). The soluble form of TREM2 (sTREM2) is generated by ɣ-secretase, which activates PI3K, extracellular signal-regulated protein kinase (ERK), and NF-kB. Src, the main effector of CSF1R, is a tyrosine kinase that phosphorylates the ITAM tyrosine residues. CCL3/4/5 binding to CCR5 activates G protein and multiple downstream signals such as Src, PLC-ɣ and PI3K. PI3K triggers the tyrosine phosphorylation of focal adhesion complex components such as Pyk2, paxillin, Crk, and p130Cas, leading to interaction with Vav. Pyk2-Vav interaction may control Rho family GTPase activation, thereby driving localized actin polymerization that stabilizes contacts with matrix and/or promote migration

Osteoclasts and microglia diverge during development and differentiation according to their tissue microenvironments. Nonetheless, they share key signaling pathways of which three receptor signaling pathways—i.e., TREM2/DAP12, CSF1, and CCR5—that converge to regulate actin-microtubule dynamics and cytoskeleton organization through Pyk2 signaling pathway (Fig. 2).

### TREM2/DAP12 signaling pathway

TREM2 is a transmembrane glycoprotein of about 40 kD with a single extracellular immunoglobulin-like domain, a transmembrane domain and a short cytoplasmic tail without signaling motif.^41^ The cytoplasmic domain of TREM2 does not have signaling motifs, therefore its transmembrane domain interacts with the adaptor protein DAP12. DAP12 is an adaptor protein that contains immunoreceptor tyrosine-based activation motif (ITAMs) in their cytoplasmic domain, which function as docking sites for protein ligand.^42^ The ITAMs of DAP12 are phosphorylated with TREM2 ligand binding, and subsequently recruit spleen tyrosine kinase (SYK) that initiates activation of various signaling cascades such as phosphoinositide 3-kinase (PI3K), VAV (Vav guanine nucleotide exchange factor), and mitogen-activated protein kinase (MAPK) activation and calcium mobilization.^42^ The ectodomain of TREM2 undergoes sequential cleavages by α-secretase and metalloprotease domain-containing protein 17 (ADAM17), which produces soluble TREM2 (sTREM2).^43,44^ DAP12 is released once the remaining C-terminal portion of TREM2 is cleaved by γ-secretase. The ITAM is rapidly phosphorylated Src tyrosine kinase, thus allowing a docking site for the SH2 domains of other kinases, such as Syk.^42^ TREM2 is expressed by osteoclasts and microglia in vivo as well as monocyte-derived DCs and bone marrow-derived macrophages for both human and mouse.^45^

#### TREM2/DAP12 mediates osteoclasts differentiation and function

Osteoclastogenesis and bone remodeling are controlled by TREM2/DAP12.^46,47^ The mechanisms underlying bone phenotype in NHD are not fully understood yet while mutations in TREM2/DAP12 are likely to disable osteoclasts to differentiate and migrate. TREM2, together with DAP12, recruits the protein tyrosine kinase Syk. Phosphorylation of Src, PI3K, and PLC-ɣ leads to Ca^2+^ mobilization and activation of the transcription factors—NFAT, NF-kB, and AP1—for osteoclast differentiation.^48,49^ ITAM-harboring adaptors FcRγ and DAP12 are essential for osteoclast differentiation through activation of Ca^2+^ oscillations and NFATc1 production in RANKL-induced osteoclastogenesis.^49^ Consistent with these findings, blockade of TREM2 inhibited bone resorption and TREM2 stimulation enhanced migration of mature osteoclasts that were generated from bone marrow macrophages or RAW264.7 cells.^50^ Additionally, human studies showed that loss-of-function variants in *TREM2* or *DAP12* result in a defective osteoclasts differentiation in response to RANKL, which cause the reduction of bone resorption ability in vitro.^51,52^ F-actin was significantly decreased in *TREM2*-deficient osteoclasts compared to wildtype.^51,52^ Proper polarization of F-actin is required for promoting cell fusion as well as transforming osteoclast precursors into multinucleated osteoclasts. Although the bone resorption capability of TREM2-/DAP12-deficient osteoclasts was impaired in vitro, the bone of NHD patients is osteoporotic due to the loss of trabeculae bone. Given these pathologic discrepancies between genetically deficient/cultured osteoclasts in vitro and local osteolytic phenotype in NHD patients, the role of TREM2/DAP12 in regulating osteoclastogenesis could be context specific. One possibility is that enhanced bone loss in vivo can be explained by systemic or local factors (endocrine, paracrine, etc.) affecting the differentiation or activation of osteoclasts in situ. A similar osteoporotic phenotype in NHD observed in *Trem2*-deficient mice.^53^ Alternatively, TREM2/β-catenin pathway regulated bone mass by modulating the rate of osteoclastogenesis.^53^ Deletion of either TREM2 or β-catenin inhibits the CSF-1-induced proliferation of osteoclast precursors but accelerates their differentiation into mature osteoclasts, which ultimately causes osteoporotic phenotype like NHD. Understanding the role of osteoclasts in the bone phenotype in NHD is yet to be elucidated while TREM2/DAP12 pathway is an attractive target for treatment of osteoporosis.

#### TREM2/DAP12 mediates microglia function

In brain, TREM2 and DAP12 are mainly expressed in microglia, but not in astrocytes.^54^ *TREM2* is actively transcribed in microglia at homeostasis. Immunogenic molecules such as lipopolysaccharides and interferon-γ suppress *TREM2* expression.^55^

Damaged neurons increase the expression of endogenous TREM2 ligands, which induces phagocytic activity by microglia.^56^ In AD brain, TREM2 is detected in neuronal and microglial cells in the vicinity of Aβ-containing plaques that leads to phagocytosis of neuronal cells with Aβ-containing plaques,^57^ which most possible is sTREM2 attached on neuronal cells. The functional deficit of TREM2, DAP12 or both leads to a failure of Aβ engulfment. As such *Trem2* deficiency augments Aβ accumulation is due to dysfunctional responses of microglia: failing to form cluster around Aβ and becoming apoptotic rather than undergoing activation and proliferation.^58^ Disease-associated microglia (DAM) are localized with Aβ-containing plaques in 5XFAD mice that express human *APP* and *PSEN1* transgenes with five known AD-linked mutations. The microglia from AD mice showed up-regulation of *Apoe, Tyrobp*, and *Trem2* and downregulation of homeostatic genes such as *P2ry12* and *Cx3cr1*.^59^ Once microglia are exposed to Aβ containing plaques, TREM2-APOE pathway is activated to transform a subset of homeostatic microglia to DAM.^60^ In summary, TREM2/DAP12 pathway is required for the recruitment and phagocytic activity of microglia in AD brain.

### CSF1 receptor signaling pathway

Colony-stimulating factor-1 (CSF1) is an essential factor that stimulates the proliferation and differentiation of macrophages from its progenitors. The expression level of CSF1 is low in HSCs and high in monocytes, tissue macrophages, osteoclast, myeloid DCs^61^, and microglia.^62^ CSF1 receptor (CSF1R, also known as c-FMS) has five immunoglobulin domains, a transmembrane domain, an intracellular juxtamembrane domain, and an intracellular tyrosine kinase domain.^63^ CSF1 binding initially leads to rapid dimerization of the CSF1R, and autophosphorylation of specific tyrosine residues and then transphosphorylation of several other proteins including SRC, PLC-γ, PI3K, AKT, and ERK in cytoplasmic tail of CSF1R.^64^ CSF1 stimulation enhanced the actin cytoskeleton reorganization and adhesion formation through integrin signal activation in macrophages.^65^

#### CSF1 stimulates the osteoclast proliferation and adhesion

Osteoclastogenesis depends on CSF1 and RANKL that are produced by osteoblasts and osteocytes. In the bone marrow microenvironment, CSF1 primarily promotes the proliferation and survival of osteoclast precursors. *Csf1*^*op/op*^ mice that express nonfunctional CSF1 lack osteoclasts and develop severe osteopetrosis. The administration of soluble CSF1 to *Csf1*^*op/op*^ mice rescues osteoclast formation ability and results in osteoporosis instead of osteopetrosis.^66^ The functional relationship between Csf1 and its receptor was established in mice lacking c-Fms (or Csf1r), which also have osteopetrosis. These mice had decreased number of macrophages in bone marrow and presented severe osteopetrosis owing to the lack of osteoclasts.^67,68^ Dimerization of CSF1R which initiated by CSF1 binding activate its tyrosine kinase domain followed by phosphorylation of six tyrosine residues in the cytoplasmic domain. These residues facilitate as high-affinity binding sites for Src homology region 2 (SH2) domains within cytoplasmic region. In turn, phosphor-Y599/c-Src recruits PI3K to activate the Akt^69^ pathway and also recruit c-Cbl E3 ubiquitin ligase complex^70^ that also regulates the proliferation of osteoclast precursors.^71^ Moreover, integrin signaling in osteoclast associated with Src is mediated by ITAM proteins including DAP12 and FcRγ, thus organizing the osteoclast cytoskeleton.^72^

#### CSF1 controls microglia homeostasis in brain

Signaling through the CSF1R is required for microglia homeostasis during development and in mature brain.^73^ Microglia are depleted in the brain of *Csf1r*-deficient mice from birth and at all stages of postnatal development.^74,75^ However, the brain phenotype caused by the loss of Csf1 signaling seems to be mild such as sensory deficit and electrophysiological abnormalities in neuronal cells.^76^ The discovery of interleukin 34 (IL-34) as a ligand for CSF1R explained, in part, the discrepancy between phenotypes of *Csf1* knock-out and *Csf1r* knock-out mice.^77^ Dominant-negative mutations in the tyrosine kinase domain of *CSF1R* causes hereditary diffuse leukoencephalopathy with axonal spheroids (HDLS) that is characterized by a broader range of neurological symptoms including progressive cognitive decline, motor disturbances and seizures.^78^ Also, CSF1 deficiency resulted in severe cerebellar phenotype such as defects in motor function and social behavior.^79^ The CSF1 receptor antagonist JNJ-40346527 is in phase I clinical trial for individuals with mild cognitive impairment (ClinicalTrial.gov NCT04121208).^80^ Importantly, this trial is designed to identify biomarkers of CSF1 signaling pathway such as IL-34 and CSF1 that may be changed in response to CSF1 receptor antagonist.

### CCR5 signaling pathway

CCR5 is belonging to the G-protein coupled receptor (GPCR) superfamily. Members of Rho family of GTPases (Cdc42, Rac1, Rac2, and RhoA etc.) are important components of signal transduction for the actin cytoskeletal rearrangement in chemokine-mediated cellular events. CCR5 regulates chemotaxis through interactions with MIP-1alpha (CCL3), MIP-1beta (CCL4), and RANTES (CCL5).^81^ Binding of CCL5 to CCR5 initiates conformational changes in G-proteins, in turn, activates multiple signaling cascades such as RAC1 and PAK2 in a G*i*- and PI3K, protein kinase C, MAPK, Pyk2, and arrestin pathways as well as calcium influx.^81–83^ These signaling pathways stimulate various cellular functions including cytoskeleton rearrangement, and chemotaxis.

#### CCR5 regulates osteoclast function

Epidemiological studies suggest that CCR5-*Δ*32 variant is associated with a reduced incidence and severity of bone destruction disease such as rheumatoid arthritis.^84^ Inhibition of *Ccr5* in mice decreased osteoclast formation suggesting beneficial skeletal effects of the functional loss of Ccr5.^85,86^ Blocking of CCR5 using antibodies impaired human osteoclast function in vitro.^87^ *Ccr5* deficient (*Ccr5*^−*/−*^) mice were less susceptibility to osteoporotic stimulation via the administration of RANKL, which induces osteoporosis. In vitro, *Ccr5*^–*/–*^ osteoclasts were showed defective actin ring formation. Also, *Ccr5*^−*/−*^ mice showed disorganized cellular motility, which was associated with reduction in the RANKL-induced phosphorylation of Src, Pyk2, and subsequent downstream signals. In patients with HIV-1 infection, maraviroc (a CCR5 antagonist) treatment was associated with reduced bone loss at the hip and lumbar spine compared to tenofovir disoproxil fumarate (TDF)-containing antiretroviral therapy (ART).^88^ These data suggest that CCR5 has a critical role in bone disorders through the functional regulation of osteoclasts.

#### Microglia migration and recruitment via CCR5 in AD

CCR5 is a co-receptor for HIV-I entry into microglia that are the primary targets of HIV infection in the CNS.^89^ HIV-infected patients develop a progressive dementia with motor and behavioral impairment, which affected 20%–30% of patients with AIDS until the introduction of combined ART in the mid-1990s.^90^ Of note, the inhibition of CCR5 signaling could be associated with the enhancement of learning and memory by elevating MAPK and CREB levels in hippocampus and cortical circuits.^91^ Treatment with maraviroc improved the neurocognitive test performance in patients with moderate cognitive impairment,^92,93^ which was presumably due to reduced immune activation and inflammation.

CCL5 is a potent mediator of microglia recruitment to the site of CNS inflammation. The reorganization of actin cytoskeleton and migration of microglia were promoted in response to CCL5 in adult rat microglia and a human microglial cell line.^94^ Immunohistochemical study demonstrated that CCR3 and CCR5 were present on microglia of normal and AD brains and upregulated in reactive microglia observed in AD.^95^ Moreover, an increased CCL5 level in microglia in the vicinity of Aβ containing plaques seems to reduce Aβ deposition.^96^

### PYK2-mediated actin cytoskeleton rearrangement

Pyk2 is a non-receptor tyrosine kinase and a member of focal adhesion kinase (FAK) family.^97^ Pyk2 is 65% homologous to FAK and shares a common domain structure—an N-terminal FERM domain, a protein tyrosine kinase (PTK) domain, three proline-rich regions, and a focal adhesion targeting (FAT) domain at the C-terminus—and has a SH2- and SH3-domain binding site. Although FAK is ubiquitously expressed in diverse cell types, the expression of Pyk2 is restricted to the CNS and in hematopoietic cells.^98^ Pyk2 is activated by various extracellular signals including cytokines, intracellular Ca^2+^ concentration, and integrin-mediated cell adhesion.^98,99^ FAT domain of Pyk2 is thought to interact with a paxillin and Pyk2-FAT and paxillin complex organize focal adhesion complexes and cytoskeletal rearrangement.^100^ *Pyk2*^*−/*−^ mice are viable and fertile, without disability in development or behavior.^101^ However, macrophages isolated from *Pyk2*^*–/*–^ mice were impaired to migrate in response to chemokine stimulation.^101^

The actin cytoskeleton plays essential roles for diverse cellular processes such as cell migration, axonal growth, phagocytosis and many other aspects of normal cell physiology.^102^ In immune cells, the ability to rapidly change shape in response to various stresses is critical for phagocytosis. Moreover, the cytoskeleton brings surface receptors and their substrates together to regulate signal transduction. Here we highlight that Pyk2 is a tethering mediator for actin reorganization of microglia and osteoclasts and that Pyk2 downstream pathway could be a converging point of cell receptor signaling pathways described above in the context of driving pathophysiological changes in osteoporosis and AD.

#### Pyk2 regulates osteoclastic bone resorption

Osteoclasts derived from hematopoietic precursor cells of the phagocyte lineage and differentiate into giant multinucleated cells by the fusion of osteoclast precursors.^1,103^ Mature osteoclasts have highly specialized morphological structures such as actin rings, sealing zone, and ruffled borders that construct an efficient machinery for dissolving hydroxyapatite and degrading bone matrix. Adhesion to bone matrix initiates osteoclast activation.^104^ Bone resorption is activated, the actin cytoskeletal reorganization is then regulated by a signaling network that includes integrins, the assembly and disassembly of focal adhesion proteins (paxillin, vinculin, and talin), c-Src^105,106^ and Pyk2^107^ in osteoclasts. Pyk2 is a main adherent tyrosine kinase in osteoclasts and regulates osteoclastic actin cytoskeletal organization in podosome for bone resorption.^108^ Once attached to the bone matrix, Pyk2 localizes to cytoskeletal proteins and colocalizes with the F-actin of podosomes.^109^ Pyk2 also colocalizes with vinculin in actin rings of osteoclasts when cultured on glass. Impaired bone resorption was observed in Pyk2-deficient osteoclasts due to their defection of podosome formation at the cell periphery.^110^ Moreover, Pyk2-deficient osteoclasts had significant reduction of microtubule acetylation and stability. As C-terminal domain of Pyk2 has paxillin-binding sites, Pyk2–paxillin complex is tightly associated with the recruitment of cytoskeletal proteins and the integrin activation in osteoclasts.^107^ Binding of Csf1 to its receptor, Csf1r-α_V_β_3_ integrin association regulates the podosomal actin ring of osteoclast during adhesion by the pathway involving Pyk2, p130Cas and c-Cbl that known as downstream regulators of integrin-mediated signaling.^111^ Moreover, Dap12 activates Syk, and Pyk2, which promote phosphorylation and nuclear translocation of β-catenin.^53,112^ Together, multiple studies support that Pyk2 is required for normal cytoskeletal organization in osteoclasts for bone resorption.

#### Dysregulation of microglia function through Pyk2 signaling pathway in AD brain

Microglia are highly dynamic cells that undergo rapid cellular remodeling during membrane extension, migration, and phagocytosis.^113^ These processes are orchestrated by changes in the organization of the actin cytoskeleton and focal adhesions. Upon brain injuries or under pathological conditions, microglia undergo morphological transformation from “inactive” to “active” state. In response to inflammatory signals, CCL5 can elicit a change in the organization of F-actin cytoskeleton in rat microglia and human fetal microglial cell line that drives chemotaxis.^114,115^ Binding of a chemokine ligand to its seven-transmembrane domain receptor initiates the release of intracellular second messengers via G-protein complexes. This, in turn, causes downstream effects such as the reorganization of the cytoskeleton, formation of focal adhesion, and pseudopod extension, that are required for cell locomotion.^94^ Pyk2 is closely related to p125 FAK—coupling several receptors including integrin and chemokine receptors—for which a variety of downstream effectors—e.g., small G proteins—are involved in actin reorganization events, membrane ruffling and motility.^116^ For instance, tyrosine-phosphorylated Pyk2 was rapidly upregulated in activated microglia after focal cerebral ischemia and epilepsy in rat model.^117^ Several studies demonstrated that Aβ binding to CD36 on microglia initiated signal transduction and activation. Fyn (a Src family kinase) is activated by CD36 after binding to Aβ. In turn, Fyn phosphorylates p130Cas.^113^ Then, p130Cas is associated with the Pyk2 and paxillin for regulating the microglial cytoskeletal reorganization.^96^ Therefore, these downstream cascade of CD36 highlight the importance of microglial migration via actin polymerization in AD.^118^

### Pyk2-mediated actin cytoskeleton reorganization in other immune cells

Cytotoxic T lymphocytes (CTL) are antigen (Ag)-specific cytotoxic cells, migrate to the infection area, and adhere to infected cells. Once T cells are stimulated with various ligands via T-cell receptor and integrins, and downstream Pyk2 is then activated. Pyk2 inhibition in CTL caused reduced cell motility and chemotactic difference.^119^ In a natural killer cell line (i.e., NK-92), the inhibition of Pyk2 activity decreased the integrin-regulated adhesion and defected clustering with a target cell.^120^ Activated eosinophils are recruited into infection cell and participate in inflammatory processes, such as allergic reactions.^121^ Blockade of Pyk2 using dominant-negative C-terminal Pyk2 fused to a TAT protein transduction domain (TAT-Pyk2-CT) inhibited the migration of eosinophils in a murine model of asthma.^122^ Pyk2 is activated by β_2_-integrin binding and is a required signal for eosinophil mobility and subsequent chemotaxsis.^123^ Similar to integrin ligation, neutrophil-like cells (HL-60) by silencing of Pyk2 expression were also attenuated cell migration.^124^ In DCs, interaction of gp120 with CCR5 initiated that the signal cascade of Pyk2 phosphorylation, which in turn activates p38 MAPK. p38 MAPK activates LSP1 (leukocyte-specific protein 1, F-actin-binding phospho-protein expressed in all human leukocytes), which then associates with actin, leading to consequent dendritic migration and chemotaxis.^125^ Taken together, these studies support an evidence of the Pyk2 function to actin cytoskeleton rearrangement, demonstrating that it contributes to the promotion of phagocytosis and migration in various cell type.

### PTK2B encompasses risk alleles for osteoporosis and AD

**Fig. 3:**
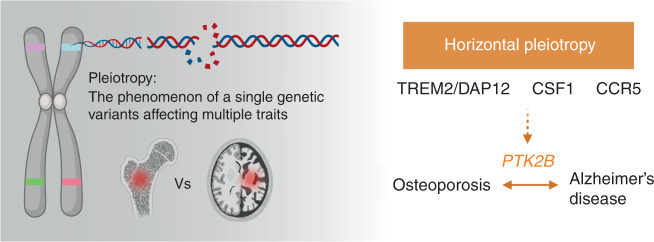
Horizontal pleiotropy of common genetic factors. Pyk2 signal could be a converging pathway of the genetic correlation between osteoporosis and Alzheimer’s disease (AD). Genetic variants in PTK2B are significantly associated with AD, body mass index, and bone mineral density suggesting that the two traits—osteoporosis and AD—could be linked by horizontal pleiotropy

Pleiotropy is the phenomenon of a single gene affecting multiple traits. In the case of NHD, rare loss-of-function variants in *TREM2* or *DAP12* result in osteoporosis and presenile dementia. Interestingly, osseous symptoms precede neurological manifestation. For this phenomenon, vertical pleiotropy can be the case where TREM2/DAP12 variants cause osteoporosis in the age of 20–30 years, which in turn causes presenile dementia in later life. Yet there is no evidence that osteoporosis causes AD. An alternative explanation can be the genetic variants in *TREM2* or *DAP12* are associated with two traits independently—i.e., horizontal pleiotropy—that are mediated by a common protein or pathway. The genetic variants in protein tyrosine kinase 2β (*PTK2B)* are significantly associated with AD, body mass index, BMD, and Takayasu arteritis suggesting that Pyk2 pathway could be a converging pathway of the genetic correlation between osteoporosis and AD (Fig. 3, Table 1).

**Table 1:** Risk alleles in three signaling pathways and PTK2B associated with bone and brain phenotypes from GWAS catalog

Common pathway	Symbol	GWAS catalog		Trait(s)		PMID		rsID	Gene name
		GWAS:BMD	GWAS:AD	Bone related	Brain related	Bone	Brain		
TREM2/DAP12	*TREM2*	No	Yes		Alzheimers disease (late onset)		PMID: 23150908	rs75932628-T	Triggering receptor expressed on myeloid cells 2
	*TYROBP*	No	No						Transmembrane immune signaling adaptor
	*SYK*	No	No						Spleen associated tyrosine kinase
	*PI3K*	No	No						Phosphatidylinositol-4,5-bisphosphate 3-kinase catalytic subunit alpha
	*SRC*	Yes	No	Rheumatoid arthritis		PMID: 30891314			SRC proto-oncogene, non-recpetor Tyrosine kinase
	*mTOR*	Yes	No	Heel bone mineral density		PMID: 28869591, PMID: 30048462		rs75077113-A	Mechanistic target of rapamycin kinase
	*RAC1*	No	No		Post-traumatic stress disorder		PMID: 31358989	rs33986000-?	Rac family small GTPase 1
	*VAV3*	Yes	No	Heel bone mineral density	Anxiety disorder/bipolar disorder/PHF-tau measurement	PMID: 30595370	PMID: 31043756, PMID: 32450446, PMID: 26989097	rs10881475-?, rs436129-G, rs1777451-?, rs10881475-?	Vav guanine nucleotide exchange factor 3
	*PTPN6*	No	Yes		Schizophrenia		PMID: 27846195	rs7963446-?	Protein tyrosine phosphatase non-receptor type 6
CSF1/CSF1R	*CSF1*	Yes	No	Bone density/heel bone mineral density		PMID: 29304378, PMID: 30595370		rs7548588-T, rs7364724-A	Colony stimulating factor 1
	*CSF1R*	No	No		Feeling emotionally hurt measurement		PMID: 29500382	rs2027798-T	Colony stimulating factor 1 receptor
CCR5	*CCR5*	No	No						C-C motif chemokine receptor 5
	*CREB1*	No	No						cAMP responsive element binding protein 1
	*PTK2 (FAK)*	No	No		Cognitive function/neurociticism /brain volume		PMID: 32439900, PMID: 29500382, PMID: 31676860	rs10106406-C, rs6997840-T, rs1326108-?	Protein tyrosine kinase 2
	*CDC42*	Yes	No	Heel bone mineral density	Intelligence/cognitive function/Schizophrenia	PMID: 30048462	PMID: 29844566, PMID: 31374203	rs10917152-T, rs5772984-?, rs2143103-A, rs2143103-?	Cell division cycle 42
	*RHOA*	Yes	No	Vitamin D measurement	Intelligence/congnitive function	PMID: 32059762	PMID: 29942086, PMID:30038396	rs7623659-T	Ras homolog family member A
	*ACTR2*	Yes	No	Heel bone mineral density		PMID: 30598549, PMID: 30048462		rs36010930-T, rs4358110-?	Actin related protein 2
Pyk2	*PTK2B*	Yes	Yes	Bone mineral density/Takayasu arteritis/bone mineral content	Alzheimer’s disease (late onset)	PMID: 31790847, PMID: 25604533	PMID: 24162737, PMID: 30617256, PMID: 29777097, PMID: 31473137	rs28834970-C, rs2271920-A, rs7000615-C, rs2322599-G, rs7005183-?	Protein tyrosine kinase 2 beta

Autosomal dominant AD is caused by one of the mutations in *APP*, *PSEN1*, or *PSEN2*. Rare genetic variants in these genes have high impact; however, such causal variants are not found in the majority of individuals with late-onset AD. A large-scale meta-analysis of 74 046 individuals highlighted novel susceptibly loci for AD.^126^ In addition to the *APOE* locus, this study discovered 11 novel loci that were significantly associated with AD. Among these, rs28834970 in the *PTK2B* gene is associated with the increased risk of AD (OR 1.10, 95% CIs 1.08–1.13, corrected *P* value 7.4 × 10^−24^). The *PTK2B* gene encodes Pyk2 that is a key regulator of converging pathways between osteoclasts and microglia as described above. The association has been replicated in later studies.^127^ Moreover, an unbiased association study using all UK Biobank traits discovered the association between another variant in *PTK2B* gene (rs7000615) and BMD (*P* value 7 × 10^−8^).^128^ The heritability of BMD was reported as 0.50–0.85 based on twin and family studies.^129^

The genetic correlation between osteoporosis and AD shall be polygenic. Using published GWAS results, we checked if there exists shared genetic risk between osteoporosis/BMD and AD by calculating polygenic risk scores (PRSs) for the two traits in population data and testing correlation between the PRSs from two traits. To calculate PRS for osteoporosis and AD, we used the GWAS summary statistics published by UK Biobank (http://www.nealelab.is/uk-biobank) for phenotype codes “20002_1309” (Non-cancer illness code, self-reported: osteoporosis) and “AD”. For 2 504 individuals from the phase 3 release of the 1 000 Genomes project, we calculated PRSs for osteoporosis and AD using the summary statistics. We did not observe significant correlation between the PRSs for osteoporosis and AD across individuals (*r*^2^ = −0.023, *P* value of 0.605 8 for Europeans). Therefore, the genetic risks due to common variants for two conditions are likely independent to each other. Alternatively, some risk loci could increase the risk for one condition but decrease the risk for the other and inter-individual variation in genetic susceptibility to environmental risk factors (i.e., gene–environment interactions) exists. Finally, two conditions may not share the majority of the genetic risks due to common variants except for those in *PTK2B*.

## Concluding remarks

Discovering diagnostic and treatment biomarkers for neurodevelopmental and neurodegenerative disorders is challenging, in part, due to limited accessibility to directly affected tissue and cell types in human. Early diagnosis of neurodegenerative disorders could change clinical course and outcome; however, clinical suspicion is often delayed until overt signs and symptoms are severe enough to get attention. Aβ deposition starts 15–20 years before cognitive symptom appears, which suggests time window for effective disease-modifying treatment for AD shall be much earlier than current practice and clinical trials.^15^ Clinical manifestation of distant organs such as BMD changes precedes later-onset AD in some cases. In NHD, mutations in TREM2/DAP12 cause bone disorder followed by brain phenotype. There are shared pathobiological mechanisms involving several molecular pathways between bone and brain although the origin of pleiotropic effects is yet to be elucidated. Here we reviewed the signaling pathways important for osteoclasts and microglia, and highlighted the convergence of these pathways to the regulation of actin cytoskeleton remodeling via Pyk2 pathway. As genetic variants in *PTK2B* increase the risk of osteoporosis and AD, Pyk2 pathway may be eligible for horizontal pleiotropy and suggest novel diagnostic and treatment biomarkers for AD. With the availability of medical big data from electronic health records, longitudinal analysis of diverse clinical phenotype for each individual can reveal association between diseases affecting distant organs. This type of analysis is complementary to the framework of case-control comparisons including GWAS although statistical approach and power analysis need to be established for longitudinal medical data. For individuals with osteoporosis, preemptive genotyping for TREM2/DAP12, CSF1, CCR5, and Pyk2 signaling pathways could suggest relative risks for AD compared to population norm with a matched genetic background. As early detection and pharmacological intervention are the only effective treatment for AD, careful evaluation and follow-up for individuals with osteoporosis can modify clinical outcome at least in a subgroup.
